# Electrostatic Protein–Polysaccharide Assembly as a Potential Alternative to Ionic Gelation for Millimeter-Scale Hydrogel Beads: Insights into Accelerated Gelation from an Amaranth Protein–Xanthan Gum System

**DOI:** 10.3390/gels12050406

**Published:** 2026-05-08

**Authors:** María del Carmen Cortez-Trejo, Ramón Román-Doval, Lucía Abadía-García, Sandra O. Mendoza, Silvia L. Amaya-Llano

**Affiliations:** 1 School of Chemistry, Autonomous University of Querétaro, Santiago de Querétaro 76010, Mexico; mariadelcarmen.cortez@uaq.mx (M.d.C.C.-T.); lucia.abadia@uaq.mx (L.A.-G.); 2 Tecnológico Nacional de México, Instituto Tecnológico del Valle de Etla, Oaxaca 68230, Mexico; ramon.rd@itvalletla.edu.mx

**Keywords:** millimeter hydrogel beads, short-term electrostatic gelation, external acidification, extrusion, protein–polysaccharide gels, xanthan, amaranth proteins

## Abstract

Electrostatic protein–polysaccharide hydrogels are attractive materials formed without thermal denaturation or chemical crosslinkers and at low biopolymer contents. Their broader application in foods, however, has been limited by slow gelation, with network development often requiring many hours (~18 h). In this study, millimeter-scale hydrogel beads were fabricated from amaranth proteins and xanthan gum by extrusion into glucono-δ-lactone (GDL) solutions (1–5 mg/mL) using hardening times of 10 or 30 min. Beads were successfully formed under all conditions (3.07–3.95 mm diameter), and their physicochemical properties, intermolecular interactions, microstructure, and gel strength were evaluated. Electrostatic attraction remained the dominant force driving gelation. Furthermore, 10 min hardening favored interpolymeric electrostatic interactions, whereas longer exposure reduced them and promoted hydrogen bonding and hydrophobic interactions. These molecular rearrangements were accompanied by a decreased size, lower water retention capacity (WRC), and higher mechanical strength. The mildest treatment (1 mg/mL GDL, 10 min) was post-loaded with a coffee pulp phenolic extract and showed reduced gel strength and electrostatic interactions, suggesting competition for binding sites within the macromolecular network. The extrusion of amaranth protein–xanthan gum mixtures into a GDL bath markedly shortens electrostatic gelation time, supporting this approach as a potential alternative to ionic gelation for the production of millimeter-scale hydrogel beads for food applications.

## 1. Introduction

Hydrogels are three-dimensional polymer networks that can absorb and retain large amounts of water [1,2,3]. In food systems, they are widely used as structuring agents and delivery matrices because they can entrap water and bioactive compounds [4].

Hydrogels are commonly classified as chemically or physically crosslinked systems, depending on the nature of the interactions stabilizing the network. Chemically crosslinked hydrogels are formed through permanent covalent bonds, whereas physically crosslinked hydrogels are stabilized by non-covalent interactions such as hydrogen bonding, hydrophobic interactions, and ionic (electrostatic) interactions [5].

Among the second group, “electrostatic hydrogels” are systems in which network formation is predominantly mediated by attractive interactions between oppositely charged macromolecules without crosslinkers or thermal treatments and at low biopolymer concentrations (≤1% *w*/*v*) [6]. However, a major limitation of electrostatic hydrogels is their prolonged gelation time, which can extend up to ~18 h, thereby limiting their industrial applicability and motivating the development of faster structuring strategies.

The formation of electrostatic hydrogels in protein–polysaccharide systems is largely controlled by environmental conditions such as pH, which determine charge density and interaction potential. At an appropriate pH, typically between the protein’s isoelectric point (pI) and the polysaccharide’s pKa, the oppositely charged biopolymers can interact to form electrostatic junction zones that stabilize the gel network. In addition, gelation occurs only under slow acidification and quiescent conditions [6]. To induce slow, homogeneous, controlled acidification, glucono-δ-lactone (GDL) is preferred, as it slowly hydrolyzes to gluconic acid and is a safe, widely used acidulant in the food industry [7].

Only a limited number of true electrostatic hydrogel systems, really formed without heating or chemical crosslinkers, have been reported. One representative example is β-lactoglobulin–xanthan gum mixtures, where internal slow acidification with GDL induced the formation of viscoelastic gel networks, whose strength and water-binding behavior depended on the balance of intermolecular interactions [8,9]. In this system, the gradual release of gluconic acid from GDL into the biopolymer mixture under quiescent conditions led to a controlled decrease in pH, which promoted the formation of oppositely charged zones within the biopolymers and facilitated electrostatic interactions, accompanied by structural rearrangements that enabled network formation [6].

More recently, amaranth protein–xanthan gum systems have been proposed as a promising plant-based electrostatic hydrogel system. Amaranth (*Amaranthus* spp.) is a pseudocereal with a relatively high protein content and a well-balanced amino acid profile, particularly rich in lysine, which is often limiting in conventional cereals. Beyond their nutritional value, amaranth proteins are mainly composed of globulins and albumins, which contain cationic amino acid residues such as arginine, lysine, and histidine; exhibit relatively high solubility and aggregation behavior [10,11]; and play a key role in determining their compatibility with polysaccharides [12].

A previous investigation by our group demonstrated that electrostatic interactions between amaranth proteins and xanthan gum can occur, as confirmed by zeta potential measurements showing pH-dependent charge complementarity between the two biopolymers [13]. Furthermore, under slow acidification with GDL, amaranth protein–xanthan gum mixtures were able to undergo electrostatic gelation [14,15].

Xanthan gum is a high-molecular-weight anionic polysaccharide produced by *Xanthomonas campestris*, widely used in food systems for its thickening and stabilizing properties. Structurally, it consists of a cellulose-like β-(1→4)-glucan backbone with alternately charged trisaccharide side chains containing mannose and glucuronic acid residues, which confer a strong polyelectrolyte character [16]. It is stable over a broad pH range and possesses a strong interaction capacity with oppositely charged protein patches [6].

Compared with whey protein-based systems, plant protein-based electrostatic hydrogels are gaining relevance owing to their sustainability and the increasing demand for plant-derived ingredients in food applications [17]. In this context, amaranth–xanthan gum systems show promising potential; however, they have primarily been studied as bulk hydrogels, in which network development requires many hours, limiting their practical application.

Most food hydrogels are commonly produced as bulk gels, although they can also be fabricated as films or beads [1,2,3]. Hydrogel beads are spherical, three-dimensional gel particles in which active materials can be dispersed or embedded in a core surrounded by a continuous protective matrix [18], typically ranging from 100 nm to several millimeters in size. In particular, millimeter-scale hydrogel beads have attracted considerable interest due to their suitability for delivering bioactive compounds [19,20]. In foods, hydrogel beads may offer advantages over conventional encapsulation systems owing to their safety, targeted delivery capacity, and ease of fabrication, as they can generally be produced without specialized or costly equipment [18].

The most widely used technique for producing millimeter-scale hydrogel beads is extrusion [18], originally developed for external alginate ionic gelation, where droplets of alginate solution are dripped into a calcium chloride bath to induce rapid crosslinking by Ca^2+^ ions [21,22,23,24]. However, this approach is largely limited to ionic polysaccharides capable of forming ionically crosslinked networks, such as alginate, agar, chitosan, and carrageenan [18].

More recently, an external acid-triggered gelation approach has emerged. Tangsombun et al. [23] demonstrated the formation of supramolecular gels based on synthetic low-molecular-weight gelators and gellan, where gel formation was initiated by protonation and progressed as the acid diffused through the material. In this context, the extrusion of protein–polysaccharide mixtures into an acidifying bath (employing a food acidulant such as GDL) may represent a strategy to accelerate electrostatic gel formation while enabling the fabrication of millimetric hydrogel beads. We expect that, upon contact with the acidified medium, proton diffusion will drive network formation by decreasing pH from the droplet surface to its core and enabling electrostatic interactions between polymers. Furthermore, the resident time and acid concentration in the gelling bath are expected to affect the gelation process and to substantially affect key functional properties, such as mechanical resistance and water retention capacity (WRC).

Despite its potential relevance to structured food design and encapsulation technologies, the production of millimeter-scale electrostatic hydrogel beads based on protein–polysaccharide systems remains poorly explored, and, to the best of our knowledge, no studies have reported the fabrication of such gels under externally acidified conditions within short timescales. Therefore, this study aims to explore the feasibility of fabricating millimeter-scale hydrogel beads from amaranth protein–xanthan gum mixtures via extrusion into a GDL bath, providing initial insight into electrostatic gelation under externally acidified conditions. This approach is proposed as a potential alternative to conventional ionic gelation for millimeter-scale bead production, relying on protein–polysaccharide electrostatic interactions induced by acidification. In this context, the effects of GDL concentration and hardening time on intermolecular interactions, microstructure, mechanical properties, and water-related behavior were evaluated. Understanding these relationships may help reduce the practical time required for electrostatic gel formation and expand the range of biopolymers suitable for millimetric bead fabrication.

## 2. Results and Discussion

### 2.1. Macroscopic Observation, Size, and pH

Hydrogel beads were produced under all experimental conditions (Figure S1a). In our previous studies [14,15], bulk APC-XG electrostatic gels were formed by internal acidification with GDL, and network development required ~18 h, as previously mentioned. In contrast, in this work, discrete hydrogel beads were obtained within 10–30 min in an externally acidified system. Although the two systems are not strictly comparable due to differences in acidification, this observation suggests a substantial reduction in the practical time required for electrostatic gel formation.

APC-XG hydrogel beads ranged from 3.07 to 3.95 mm (Table 1). Beads formed at GDL concentrations of 1 and 2.5 mg/mL exhibited significant differences in size at both hardening times (*p* ˂ 0.05); as the GDL concentration increased, bead diameter decreased. However, no significant differences were observed between the 2.5 and 5 mg/mL GDL at any time. The duration of the hardening process significantly influenced bead size across all tested GDL concentrations; longer hardening times resulted in smaller beads (*p* < 0.05).

A two-way ANOVA, on the other hand, showed that both hardening time and the interaction between the two factors had a significant effect on bead size (*p* < 0.05), whereas GDL concentration alone did not have a significant global effect (Table S1). Pairwise comparisons revealed differences between certain GDL levels, such as 1 and 2.5 mg/mL. However, since there was no clear trend across all concentrations, this suggests that GDL’s effects vary and depend on its interaction with hardening time.

In externally gelled bead systems, gel formation is driven by the diffusion of the gelling agent (protons from gluconic acid, released from GDL in this study) and progresses from the droplet interface toward the interior [25]. At the same time, as the three-dimensional network is forming, the amount of water retained within it is predominantly determined by osmotic pressure and the elasticity of the polymeric gel matrix [26]. Both higher GDL concentrations and prolonged hardening times rendered the hydrogel beads more restrictive to water molecules, possibly explaining the observed decrease in size.

It is noteworthy that, although the molecular basis of APC-XG electrostatic gelation and ionic (alginate) gelation differs, an analogy can be drawn at the level of the extrusion methodology: In both systems, gelation is induced by the diffusion of species from the surrounding medium into the droplet. In ionic gelation, Ca^2+^ ions diffuse into the droplet and function as crosslinkers, forming ionic bridges between negatively charged polymer chains. In contrast, in the present system, protons from the acidified GDL bath are expected to diffuse into the droplet, progressively altering the ionization state of the biopolymers. Amino acids in proteins are protonated and generate positive charges, while protons can also modulate the degree of ionization of carboxylate xanthan gum. As a result, oppositely charged sites can electrostatically interact, driving network formation.

In ionic systems, an increase in Ca^2+^ ions within the gelling bath has been reported to induce rapid gelation, thereby reducing the size of alginate hydrogel beads [27]. In a similar way, in the present electrostatic systems, the gluconic acid released from GDL into the gelling bath may lead to observed trends in bead size, despite fundamentally different gelation routes.

As a general comparison, the APC-XG hydrogel beads fell within the reported size range (1–8 mm) for millimeter-scale beads produced by the ionic gelation of alginate [28], gelatin–carrageenan [29], alginate–chitosan [30], and alginate–chitosan/gelatin mixtures [31].

The final pH values of the hydrogel beads, on the other hand, are also shown in Table 1. All treatments yielded beads with pH values below the reported pI (~4.9) for APC [13]; thus, electrostatic interactions between positively charged amaranth proteins and negatively charged XG molecules were expected, as confirmed by intermolecular force and FTIR analyses.

Considering that the initial pH of the APC-XG mixture was 10 and dropped to 3.0–4.5 in the beads, the acidification process was evident. This is consistent with a diffusion-driven process, in which proton transport from the surrounding medium dictates the spatial and temporal evolution of network formation during hardening, as reported in some synthetic hydrogel systems where acid diffusion generates a pH gradient that controls gelation [25,32].

pH is recognized as the primary factor influencing electrostatic interactions and compatibility within protein–polysaccharide systems [33,34], since biopolymer properties such as solubility, molecular configuration, and charge density are pH-dependent [24]. pH directly controls the protonation state of ionizable groups in biopolymers.

Amaranth proteins contain significant amounts of basic amino acids, including lysine, arginine, and histidine [10], which acquire a positive charge under acidic conditions. Lysine and arginine, in particular, are protonated over a wide pH range due to their high pKa values (10 and 12, respectively [35]). In contrast, xanthan gum contains carboxyl groups that are deprotonated above their pKa (approximately 3 [36]). In a thermodynamically compatible scenario, oppositely charged sites along the polymer chains enable electrostatic attraction between positively charged protein regions and negatively charged xanthan chains, as previously demonstrated by zeta potential measurements in APC-XG systems [13]. Then, interpolymeric complexes can form, and, under appropriate conditions of total biopolymer content and protein–polysaccharide ratio, the structured system further evolves into an electrostatically interconnected, three-dimensional network [6].

### 2.2. Intermolecular Forces and Secondary Structure Analysis

**Figure 1:**
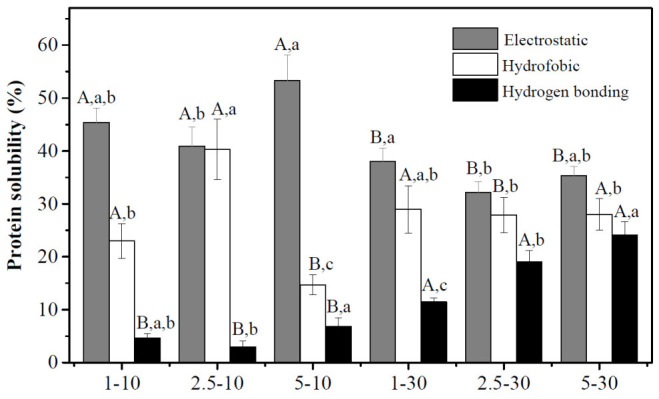
Intermolecular forces in APC-XG hydrogel beads. Uppercase letters show statistical comparison (*p <* 0.05) among hardening times at the same GDL concentration for each type of interaction. Lowercase letters indicate comparison between GDL concentrations at the same time for each type of force. APC, amaranth protein concentrate. GDL, glucono-δ-lactone. XG, xanthan gum.

A study of protein solubility in hydrogel beads across four solvents was conducted to determine the intermolecular forces responsible for gel crosslinking. Figure 1 shows that, across all treatments, the highest protein solubility values corresponded to electrostatic interactions (32–53%), confirming the electrostatic nature of the APC-XG beads. In all cases, hydrophobic interactions were the second most important force, followed by hydrogen bonding. These observations align with those of our previous study on the same APC-XG system (1:1 ratio and 1% total biopolymer content), which showed that internal acidification with GDL enabled electrostatically driven gelation, with an electrostatic contribution of ~50% [14].

In this study, the use of external acidification imposed additional constraints on the electrostatic gelation process. A two-way ANOVA showed that both GDL concentration and hardening time had significant effects on electrostatic interactions (*p* < 0.05), with no significant interaction between the factors (Table S1), indicating that their effects act independently as acidification and restructuring progress over time. However, mean comparisons revealed that the influence of GDL concentration was not uniform across conditions. After 10 min of hardening, an increase in electrostatic interactions was observed at GDL concentrations between 2.5 and 5 mg/mL (*p* < 0.05), whereas after 30 min, a significant reduction from 1 to 2.5 mg/mL was detected (*p* < 0.05). This behavior suggests that, although GDL concentration affects electrostatic interactions, its impact depends on the stage of structural evolution rather than following a consistent trend.

Contrary to expectations, electrostatic interactions decreased with increasing hardening time at all three GDL concentrations. This may be related to the formation of new hydrophobic interactions and hydrogen bonds as the bead hardening time increased from 10 to 30 min. The restriction on the biopolymers’ incorporation of water molecules, driven by the osmotic pressure of the GDL solution, could explain the formation of hydrophobic interactions between protein residues and hydrogen bonds between proteins and XG. For instance, cosolutes can significantly influence polymer aggregation in gelling systems. Oakenfull and Scott [37] evaluated the effect of several sugars (glucose, sorbitol, and fructose, among others) as cosolutes on the gelation process of pectin. The findings indicated that their presence enhanced pectin aggregation through hydrogen bonding and hydrophobic interactions by restricting water mobility. In this work, the gluconic acid released from GDL in the gelling solution likely functioned as a cosolute, in addition to its primary role as an acidifier. It may have facilitated aggregation among XG molecules, between amaranth proteins and XG via hydrogen bonding, and among proteins in the APC through hydrophobic interactions.

Another factor that could have contributed to the reduced interpolymeric electrostatic interactions observed in the 30 min hardened hydrogels is pH. These beads exhibited pH levels ranging from 3.02 to 3.56, at which the charge of XG is less negative than at higher pH values [36], as observed in the 10 min hardened gels. Consequently, pH values approaching 3 may decrease the number of negatively charged sites on XG molecules available for interaction with APC.

**Figure 2:**
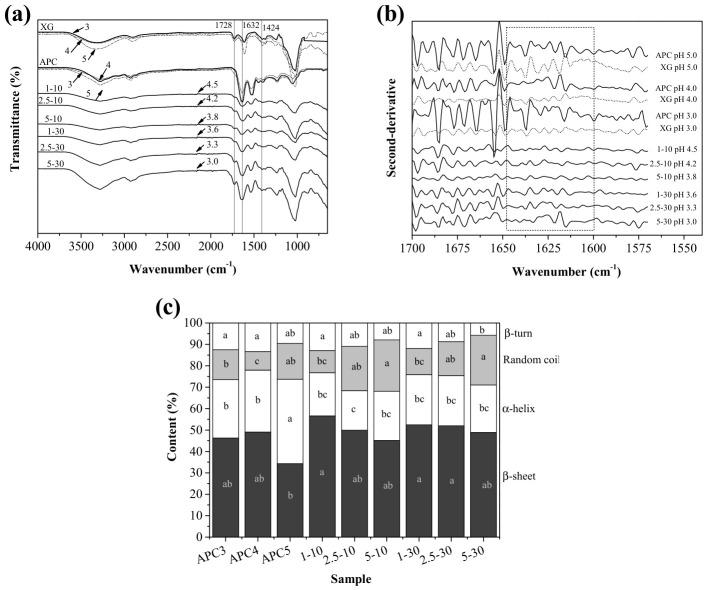
Fourier-transform infrared spectroscopy (FTIR) analysis of APC-XG hydrogel beads. (**a**) ATR-FTIR spectra of xanthan gum (XG), amaranth protein concentrate (APC), and hydrogel bead samples prepared under different GDL concentration–hardening time conditions. (**b**) Second-derivative analysis of the amide I region. (**c**) Relative secondary structure composition of hydrogel bead samples determined by deconvolution of the amide I band. The values with arrows next to each spectrum correspond to the sample’s measured pH. Distinct lowercase letters denote statistically significant differences within each structural fraction among samples (*p* < 0.05). APC, amaranth protein concentrate. GDL, glucono-δ-lactone. XG, xanthan gum.

A complementary FTIR study of the APC-XG hydrogel beads was conducted (Figure 2a) to provide molecular-level insight into the interactions between APC and XG under the specific external acidification conditions explored in this study. Individual biopolymers were analyzed within the pH range 3–5, according to the final pH values of the hydrogel beads (Table 1).

XG exhibited characteristic bands at 3317 cm^−1^ (¯OH groups; [38,39]), 2902 cm^−1^ (C-H bonding), 1728 cm^−1^ (acetyl C=O), and 1607 and 1424 cm^−1^ (COO¯ groups; [40,41]). At pH 5, all band intensities increased compared with at lower pH values, in agreement with the unfolding of the xanthan helix structure [14]. This condition may facilitate interaction with APC.

APC spectra at pH 3–5 showed similar types, numbers, and locations of characteristic bands. Among the most relevant peaks were amide I (1632 cm^−1^), associated with the C=O vibration of the peptide bond; amide II (1500–1600 cm^−1^), corresponding to the N-H vibration; and bands in the 3500–3000 cm^−1^ region, related to the ¯OH groups [42].

In the hydrogel beads, the electrostatic interaction between APC and XG, previously demonstrated by the solubility test, was confirmed across all treatments. Multiple spectral modifications indicated the formation of intermolecular electrostatic interactions, such as a reduction in intensity within the 3000–3500 cm^−1^ region, associated with diminished hydrogen bonding that favors electrostatic interactions [43]; a decrease in the band at 1728 cm^−1^, linked to alterations in the C=O group of the acetyl group, as a product of interpolymeric interactions [44]; and a reduction in the intensity of the band at 1424 cm^−1^, related to fewer available COO^−^ groups [14]. These changes were similar to those observed in the bulk APC-XG systems [14]. However, the external acidification used here was conducive to differences mainly based on hardening time. For instance, more pronounced spectral changes were observed in beads hardened for 10 min, indicating stronger interpolymeric electrostatic interactions and agreeing well with the solubility test results.

Notably, spectra from treatments 2.5–30 and 5–30 exhibited slight reductions in band intensities. Furthermore, the band in the 3500–3000 cm^−1^ region showed an increased intensity, particularly for beads 5–30, indicating that, despite prolonged exposure to GDL, the electrostatic interaction among APC and XG did not strengthen, and other types of association, especially hydrogen bonding, likely became more prevalent.

To obtain further insight into the external electrostatic gelation studied in this work, second-derivative analysis and deconvolution of the amide I region were performed (Figure 2b,c). As shown in Figure 2, beads hardened for 10 min exhibited lower intensities across nearly all resolved sub-bands than those hardened for 30 min. This effect was more marked in the 1600–1650 cm^−1^ region (dotted box in Figure 2b, where XG carboxylate group sub-bands appear (derived from the peak at 1607 cm^−1^ (COO¯ groups, Figure 2a)). The reduced intensity of these sub-bands in the 10 min treatments confirms that the functional groups in this region were more extensively involved in intermolecular electrostatic interactions between protonated amaranth proteins and xanthan carboxylate groups. Conversely, the sub-bands within this region became progressively more defined after 30 min of hardening, indicating greater spectral resolution of the original anionic groups and, therefore, a reduction in electrostatic intermolecular interactions. Indeed, the second-derivative profiles of the 30 min treatments more closely resemble those of the APC controls, suggesting that prolonged hardening reduced the extent of APC-XG electrostatic complexation and allowed greater spectral contribution from unassociated or less-associated protein functional groups. These observations agree with the findings obtained from the original FTIR spectra and the solubility test results.

Regarding the structural modifications in proteins upon bead gelation, Figure 2c shows that the distribution of secondary structures within the APC-XG hydrogel beads varied with hardening conditions and should be interpreted in light of the final pH of each treatment. Among the APC controls, a few differences were observed; however, these were generally non-significant (*p* ≥ 0.05).

A comparison of the hydrogel beads with APC controls at similar pH levels revealed only minor changes across treatments, with no clear visual trends related to GDL concentration or hardening time. However, random coil content increased with increasing GDL concentration from 1 to 5 mg/mL at both hardening times (*p* ≥ 0.05). These observations indicate that acidification may induce conformational disorder. Rapid protonation may disrupt intramolecular interactions and promote localized unfolding during gelation under externally acidified conditions.

A two-way ANOVA (Table S1) provides support for this interpretation by demonstrating that GDL concentration significantly affected random coil and β-turn structures (*p* ˂ 0.05), despite the absence of clear, consistent trends in the data. These results indicate that GDL concentration is a critical factor in inducing conformational changes, although its effects are not necessarily linear.

In contrast to previous findings on bulk APC–XG hydrogels [14], which showed no significant changes in protein secondary structure, the present results suggest that external acidification induces additional structural alterations. This effect is likely due to more rapid, heterogeneous proton diffusion during bead formation.

### 2.3. WRC, Syneresis, and Swelling Index

WRC is a key functional property of hydrogels; it depends primarily on the presence of hydrophilic functional groups within the polymeric network that enable water binding [21]. In addition, WRC is dependent on preparation conditions and network density, which determine the availability of free volume and water-binding sites within the gel structure [19].

**Figure 3:**
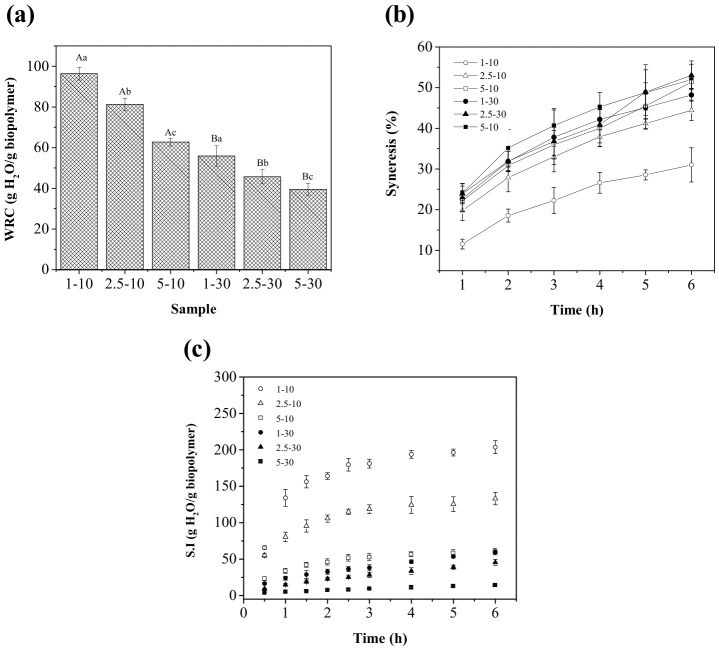
Water-related properties of APC-XG hydrogel beads produced under different GDL concentration–hardening time conditions. (**a**) Water retention capacity (WRC) of freshly prepared beads. (**b**) Syneresis percentage during 6 h of storage at room temperature. (**c**) Swelling index (S.I.) of hydrogel beads during rehydration in distilled water over 6 h. APC, amaranth protein concentrate. GDL, glucono-δ-lactone. XG, xanthan gum. Different uppercase letters indicate significant differences between hardening times within the same GDL concentration, whereas different lowercase letters indicate significant differences among GDL concentrations at the same hardening time (*p* < 0.05).

Figure 3a summarizes the WRC values of the APC-XG beads, which ranged from 39.52 to 96.49 g H_2_O/g biopolymer. The APC-XG beads retained a higher water content than several millimetric protein–polysaccharide beads produced by ionic assembly reported in the literature; for example, Bubin et al. [45] reported 15–19 g water/g biopolymer for millimetric beads of pitahaya pulp/alginate/carrageenan, while alginate and alginate/carrageenan/chitosan beads showed 11–24 g water/g biopolymer [46].

A significant effect of both hardening time and GDL concentration on WRC was observed. Increasing either variable significantly reduced WRC values (*p* < 0.05). As discussed previously, the osmotic pressure of the gelling bath may play a key role in the amount of water retained within the gel network. At higher GDL concentrations, the diffusion rate of water from the interior of the bead to the exterior is expected to be higher due to the larger osmotic pressure difference; thus, smaller beads with a lower water content were obtained. Similarly, prolonged hardening times promoted additional dehydration and contraction of the beads, further decreasing WRC. It should be noted that the treatment with 1 mg/mL GDL and a 10 min hardening time produced beads with the highest WRC value (96.40 ± 3.14 g H_2_O/g biopolymer), indicating that milder gelation conditions favored the formation of more hydrated matrices. Indeed, the WRC observed for this treatment was comparable to that previously observed for the bulk APC-XG electrostatic hydrogel (~95 g H_2_O/g biopolymer [15]), highlighting that external acidification under more intense hardening conditions affects the WRC of this electrostatic system.

Syneresis of hydrogel beads was evaluated over 6 h after fabrication at ambient temperature, and the results are shown in Figure 3b. The percentage of expelled water ranged from 11.55% to 53.02% throughout the evaluation period. Similar trends have been reported in calcium alginate hydrogel beads. For instance, Velings and Mestdagh [47] showed that alginate beads can lose up to 60% of their initial weight due to syneresis, with most water loss occurring in the first hours of storage.

Consistent with the WRC results, hydrogel beads 1–10 showed the lowest syneresis values, indicating superior water-holding capacity. Syneresis is closely related to the elasticity of the gel matrix, as the degree of crosslinking strongly influences the liquid-holding capacity of hydrogels. At higher crosslinking densities, the network exerts greater pressure on the entrapped liquid, reducing swelling and favoring liquid expulsion [26]. Astuti et al. [48] observed that increasing crosslinking conditions led to greater syneresis in iron–alginate beads, suggesting that more compact networks promote a wider range of syneresis, from moderate to very high levels, depending on the gelation conditions.

In the present study, the final pH of the APC-XG beads varied with the processing conditions, thereby affecting the intermolecular interactions involved in network stabilization. As previously discussed, beads hardened for 30 min showed greater hydrogen bonding, which may help explain their increased syneresis, as more compact networks with fewer available sites for water retention were formed.

On the other hand, the syneresis curves showed a progressive increase over time, with the highest rate of water release occurring during the initial stages of the test, followed by a slower increase thereafter. This behavior suggests that water release was more favorable during the early stages of storage and gradually decreased as the remaining water became more strongly bound with the gel matrix. Similar time-dependent syneresis profiles have been reported in hydrogel systems, where solvent expulsion occurs progressively due to gel contraction, further suggesting that syneresis may involve network rearrangement-induced transitions of water from more bound to free states prior to migration through the gel matrix [49].

The swelling index (S.I.) is another important property frequently evaluated during the fabrication of hydrogel beads, especially for encapsulation purposes, as swelling/shrinkage is considered one of the major mechanisms determining compound release in pH-sensitive protein–polysaccharide hydrogels [50]. The swelling index (or swelling ratio) of hydrogels is defined as the ability of a polymeric network to absorb and retain water, commonly expressed as the relative increase in mass or volume of the gel in its hydrated state compared to its dry state [51].

Physical forces and the elastic behavior of the polymer chains determine water absorption and swelling. Swelling in polymer networks involves the incorporation of water into the hydrophilic matrix through diffusion and capillary action. At the same time, the elasticity of the crosslink points allows the hydrogel to swell in response to physical stimuli [52]. As shown in Figure 3c, the swelling index of all APC-XG beads increased progressively over 6 h, with beads 1–10 showing the highest swelling capacity. After this period, the S.I. values ranged from 13.96 to 203.75 g H_2_O/g of polymer, following the same general trend observed for the WRC values. Specifically, swelling decreased as the GDL concentration and hardening time increased, indicating the formation of more compact network structures under these conditions.

Compared to whey protein–sodium alginate hydrogel beads (39–48 g H_2_O/g of polymer, [53]), beads hardened for 30 min in the present study exhibited lower swelling values. In contrast, beads hardened for 10 min demonstrated swelling values up to four times higher. These differences result from variations in the gelation mechanism, the specific biopolymer used, and the resulting network microstructure. Even though no direct comparison is valid, this provides a general overview of the APC-XG system’s resulting properties.

The swelling curves exhibited a biphasic behavior characterized by an initial rapid water uptake during the first hours, followed by a slower increase thereafter. This pattern is commonly observed in hydrogel systems. It suggests that water absorption initially occurred rapidly due to the high osmotic gradient between the partially hydrated matrix and the surrounding medium. As swelling progressed, the rate of water uptake decreased, likely due to the progressive saturation of hydrophilic binding sites and the increasing resistance of the polymeric network against further expansion. Hydrogels generally require variable periods to reach equilibrium swelling because water sorption depends on solvent diffusion through the matrix and progressive polymer relaxation during hydration [52]. Although complete equilibrium was not reached within 6 h for some treatments, the marked reduction in swelling rate at later times suggests that the APC-XG beads approached their hydration limit.

It is worth noting that beads 1–10 swelled faster and to a greater extent than those in all other treatments. Remarkably, although these beads initially contained 96.40 g H_2_O/g biopolymer after fabrication, upon reconstitution, they absorbed more than twice as much water (203.75 g H_2_O/g biopolymer). This result is particularly relevant for post-gelation loading applications, as it suggests substantial matrix expansion and water uptake capacity. Since the electrostatic gelation of APC-XG beads is induced by acidification, the incorporation of a bioactive directly into the precursor biopolymeric mixture may interfere with electrostatic complexation and hinder gel formation. Therefore, loading APC-XG electrostatic beads with bioactive compounds may be more suitable after bead formation.

On the other hand, the observed trends in water-related properties align with a progressive acidification process, where proton transport from the surrounding medium drives the spatial and temporal evolution of network formation [25].

A two-way ANOVA (Table S1) confirmed that GDL concentration, hardening time, and their interaction significantly affected the WRC and swelling index, indicating that water retention and uptake depend on the coupled effects of acidification intensity (GDL concentration) and network evolution (retention time). In contrast, syneresis was mainly affected by hardening time, with no significant interaction observed, suggesting that water expulsion is mainly promoted by time-dependent network contraction rather than by GDL concentration alone.

These results suggest that, as acidification progresses, continuous changes in the polymers’ ionization state facilitate the structural reorganization of the matrix. In particular, reduced swelling capacity and water retention are associated with the formation of more compact networks, where increased density restricts water uptake and mobility [52]. In this study, decreases in the WRC and swelling behavior indicate that this reorganization progresses with acidification, an assumption further supported by the increase in gel strength with hardening time.

### 2.4. Microstructure

**Figure 4:**
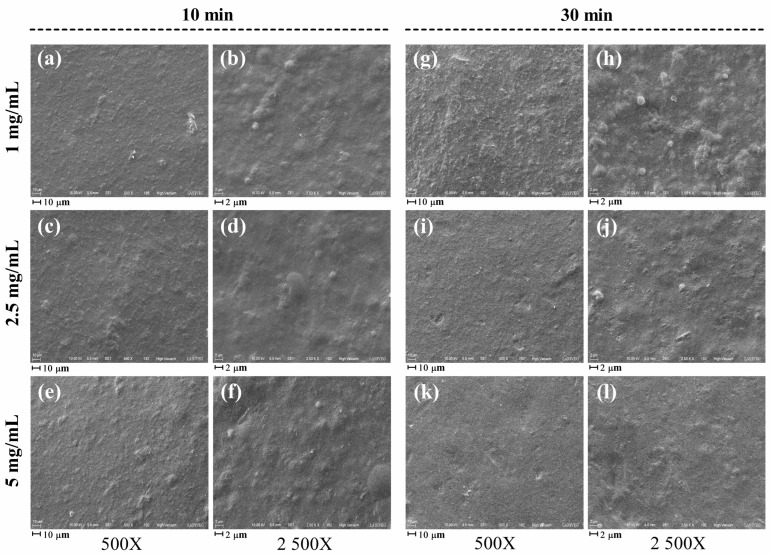
(**a**–**f**) Electron micrographs of APC-XG hydrogel beads with 10 min of hardening. (**g**–**l**) Electron micrographs of APC-XG hydrogel beads with 30 min of hardening. From top to bottom: beads fabricated with 1, 2.5, and 5 mg/mL GDL gelling solution. APC, amaranth protein concentrate. GDL, glucono-δ-lactone. XG, xanthan gum.

Figure 4 shows scanning electron micrographs of the APC-XG hydrogel beads obtained under different hardening conditions. In general, all samples exhibited heterogeneous and irregular external surfaces with rough topographies characteristic of particulate or aggregate gel networks.

Gel structure is mainly determined by the composition and gelation mechanism [24]. The observed morphology is consistent with protein–polysaccharide systems in which gelation occurs through associative interactions followed by clustering. In particular, electrostatic gels formed by proteins and xanthan gum have been reported to display aggregated structures in which protein-rich domains are associated with a polysaccharide framework [9]. Comparable microstructures were also previously observed in bulk APC-XG electrostatic hydrogels in our previous work [14].

At 10 min of hardening, slight variations in surface roughness were observed as a function of GDL concentration, which may relate to differences in the extent of electrostatic interactions between xanthan and amaranth proteins, as discussed previously. After 30 min, morphological differences became evident: the 1–30 samples exhibited more pronounced roughness and aggregation, whereas the 2.5–30 and 5–30 samples displayed relatively smoother surfaces. These observations are in general agreement with the interpretation of the intermolecular interactions derived from FTIR and intermolecular force analyses. Electrostatic interactions remained the dominant driving force for gel formation at both hardening times; however, their relative contribution increased under more intense acidification conditions. At 30 min of hardening, specifically in the 2.5–30 and 5–30 treatments, hydrogen bonding and hydrophobic interactions become more relevant, which may contribute to the development of smoother, less rough gel networks. Similar phenomena have been documented in protein–polysaccharide gels, where enhanced hydrophobic interactions and hydrogen bonding contribute to the formation of tightly packed, ordered networks [54,55].

SEM observations are based on dried samples, and the drying process may induce structural alterations. Consequently, the observed microstructure may not accurately represent the native hydrated state of the gels and should be interpreted with caution. Despite this limitation, SEM is a valuable tool for comparative analysis when preparation conditions are consistent. Future studies may employ complementary techniques, such as cryo-SEM or confocal microscopy, to further elucidate the microstructure of these systems.

### 2.5. Gel Strength

The mechanical strength of hydrogel beads is related to their resistance to bursting during processing, handling, and storage [56]. The strength values of the APC-XG hydrogel beads ranged from 1.44 to 5.80 N, with treatments 1–10 and 5–30 showing the lowest and highest values, respectively. No significant differences (*p* ≥ 0.05) were observed among the 2.5–10, 5–10, 1–30, and 2.5–30 beads, which exhibited intermediate values of 3.27–3.71 N. A two-way ANOVA (Table S1) confirmed that gel strength was significantly influenced by GDL concentration, hardening time, and their interaction. This result indicates that mechanical resistance depends on the combined effects of acidification and network evolution (hardening time), rather than on the independent contributions of each factor.

The degree of electrostatic crosslinking did not appear to be directly proportional to gel strength. Although beads hardened for 30 min exhibited lower APC-XG electrostatic interactions than those hardened for 10 min, their hardness values were equal to or greater than those of the 10 min beads. This shows that electrostatic attraction played a key role during network formation but was not the only intermolecular interaction determining the final resistance of the gel beads. Contributions from hydrogen bonding and hydrophobic interactions likely reinforced the gel matrix. Similar phenomena have been reported in protein–polysaccharide systems, where secondary interactions (e.g., hydrogen bonding and hydrophobic interactions) promoted denser, mechanically stronger structures [54,57].

Water content likely provides an additional explanation for the observed mechanical response. Beads hardened for 30 min showed a lower WRC value; their higher relative solid concentration likely contributed to greater resistance during compression. Water acts as a plasticizer in hydrogel systems; thus, lower moisture generally favors closer polymer packing and firmer textures [58]. This interpretation agrees with the highest strength observed for treatment 5–30 (5.8 N), suggesting that the highest GDL concentration and the most prolonged exposure to the gelling bath promoted matrix contraction and structural consolidation during hardening. This behavior is in line with progressive network development as acidification advances.

When comparing APC-XG beads with conventional millimetric ionic beads, the latter show stronger resistance, as expected for ionically crosslinked systems, since calcium ions form egg-box junction zones that generate stiff networks. For instance, Manev et al. [59] reported rupture forces ranging from 4.3 to 43.1 N for alginate beads containing 6% wt alginate and hardened for up to 6 h. In contrast, the APC-XG hydrogel beads fabricated in this study were stabilized mainly by electrostatic attraction, along with hydrogen bonding and hydrophobic interactions, which generally yield softer, more deformable matrices. Additionally, in this study, only 1% wt total biopolymer was utilized. Increasing the biopolymer content is known to produce stronger electrostatic hydrogels [6,8].

On the other hand, a more appropriate comparison can be made with our previous study [14]. There, the gel strength of the same APC-XG system was 345 g (~3.38 N). The hydrogel bead samples 2.5–10, 5–10, 1–30, and 2.5–30 developed comparable strength values, while the treatments 1–10 and 5–30 differed substantially (1.44 and 5.80 N, respectively). This indicates that gelating the same formulation into millimeter-sized beads by external acidification modifies, and even improves, mechanical resistance depending on the hardening conditions.

### 2.6. External Acidification and Network Evolution: An Initial Interpretation

The combined experimental evidence from the previous result sections may provide initial insights into the external acidification process that drives electrostatic gelation in the APC-XG system. Although direct kinetic or spatially resolved experiments were not conducted, the observed trends suggest a diffusion-mediated acidification pathway that connects proton transport, charge modulation, and progressive network development during millimeter-scale bead formation.

In this system, the GDL solutions were prepared 24 h in advance to allow hydrolysis to reach equilibrium and stabilize the bath pH prior to extrusion. Under these conditions, acidification of the droplets is expected to be determined by proton diffusion from the external medium into the APC-XG mixture, which initially has a pH of 10, rather than by ongoing GDL hydrolysis within the bath. Comparable diffusion-driven acidification mechanisms have been reported in other hydrogel systems, where proton transport establishes a spatially evolving pH gradient that regulates the location and progression of gelation [25,32].

As protons diffuse into the droplets, the internal pH decreases gradually, leading to continuous changes in the ionization state of both biopolymers. These changes are consistent with the previous characterization of the APC-XG system based on zeta potential measurements [13]. At alkaline pH, both APC and xanthan gum are predominantly negatively charged, which limits attractive interactions. As the pH decreases, the protein component progressively reduces its net negative charge and may reach a region where positive charges become significant, while xanthan gum remains negatively charged over a wide pH range. This creates a transient condition in which electrostatic attraction between oppositely charged species becomes favorable, contributing to the formation of a three-dimensional network [14,15].

The observed dependence of the bead size, WRC, swelling behavior, and gel strength on hardening time observed in this study can be interpreted within this framework. Short hardening times likely correspond to partial acidification, during which electrostatic interactions begin to develop, but the network may remain relatively open, allowing greater water mobility and swelling. As hardening time increases, continued proton diffusion promotes additional network restructuring. Under these conditions, reductions in swelling and WRC, along with increased gel strength, suggest the formation of a more compact structure.

As acidification progresses beyond the initial electrostatic interaction window, subsequent changes in charge density may reduce the relative contribution of electrostatic interactions. At this stage, other forces, such as hydrogen bonding and hydrophobic interactions, likely play a more relevant role in stabilizing the network. These interactions contribute to the observed increase in gel strength and decrease in water mobility.

Additional evidence was provided by the two-way ANOVA results (Table S1), which indicate significant interactions between GDL concentration and hardening time for the key parameters mentioned above (i.e., bead size, WRC, swelling behavior, and gel strength) (*p* ˂ 0.05). Thus, the observed responses result from the combined influence of both factors during the diffusion-driven acidification process. Hence, each GDL concentration, combined with the hardening time, controls the extent of this process and defines the system’s final properties.

Lastly, the preceding interpretation relies on indirect evidence obtained from macroscopic measurements and statistical analysis using a limited factorial experimental design. Although the two-way ANOVA offers initial insights, a larger, more comprehensive experimental approach is required to elucidate the underlying mechanisms. Subsequent research should directly investigate internal pH gradients, charge distributions, rheological changes, and gelation kinetics, among other relevant factors.

### 2.7. Phenolic Incorporation: Proof of Concept

To evaluate the potential of the electrostatic APC-XG beads in practical functionality as a bioactive carrier, the 1–10 treatment was selected for a proof-of-concept evaluation of phenolic incorporation. Although this treatment exhibited the lowest mechanical strength, it showed the highest swelling index, the greatest WRC, and the lowest syneresis, indicating a more hydrated network structure. Such characteristics are advantageous for encapsulation, as they may facilitate the loading of the active compound into the matrix post-gelation. Although a comprehensive characterization of the encapsulation performance was beyond the scope of this study, the results provide initial evidence of compatibility between the hydrogel network and a phenolic extract.

Hydrogel beads 1–10 loaded with a coffee pulp phenolic extract (CPE) are shown in Figure S1b. The loaded beads showed clear incorporation of phenolic compounds from the coffee pulp, as confirmed by total phenolic content (TPC) measurements (180 ± 0.01 μg GAE/g fresh beads). These results indicate that the electrostatic gel matrix can retain measurable amounts of coffee phenolics under the tested conditions, suggesting its potential for bioactive incorporation. However, the presence of the phenolic extract reduced the gel strength. While unloaded 1–10 beads exhibited a value of 1.44 ± 0.31 N, loaded beads showed a lower strength of 0.83 ± 0.10 N.

**Figure 5:**
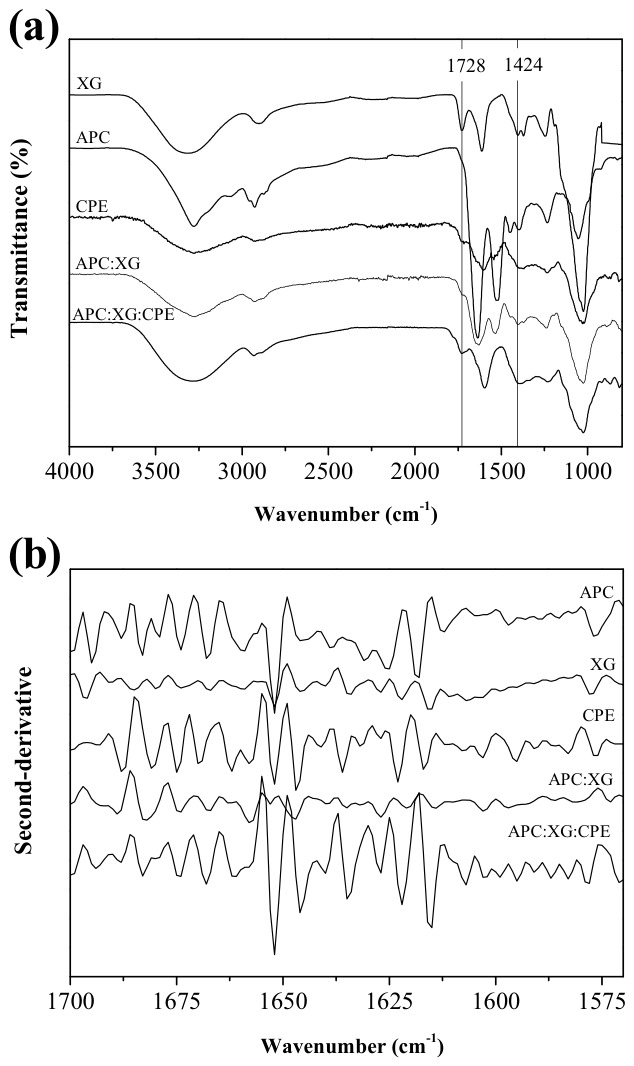
Infrared spectroscopic analysis of APC-XG hydrogel beads (1–10 treatment) before and after loading with coffee pulp extract. (**a**) ATR-FTIR spectra of individual components (APC, XG, and CPE), unloaded APC-XG hydrogel beads, and CPE-loaded APC-XG beads. (**b**) Second-derivative spectra of the amide I region showing conformational changes associated with extract incorporation. APC, amaranth protein concentrate. CPE, coffee pulp extract. XG, xanthan gum.

The rheological behavior of hydrogels depends on the strength of intermolecular interactions and the resulting network structure [60]. Thus, the compounds present in CPE likely altered the three-dimensional organization of the matrix and partially weakened the original electrostatic interactions between XG and amaranth proteins. This effect is supported by infrared spectroscopy (Figure 5a), which revealed noticeable changes between the loaded and unloaded hydrogel beads. In the loaded beads, the bands at 1424 and 1728 cm^−1^ increased in intensity, attributed to COO^−^ groups and C=O from acetyl, respectively [40,41]. The higher intensity at 1424 cm^−1^ suggests a greater proportion of free carboxyl groups that were previously involved in electrostatic interaction with amaranth proteins, whereas the change in the intensity of the peak at 1728 cm^−1^ may reflect reduced conformational rearrangements associated with interpolymeric complexation.

Additionally, the band in the 3500–3000 cm^−1^ region showed an increased intensity, indicating enhanced hydrogen bonding, possibly involving phenolics and matrix biopolymers. These observations suggest that coffee phenolics may partially replace protein–polysaccharide electrostatic junction zones with new hydrogen-bonding interactions, thereby enabling incorporation but simultaneously softening the gel network. Such behavior is common in polyphenol–biopolymer systems, where phenolics can modify protein conformation and compete for binding sites [61].

Although differences in formulation and gelation mechanism limit direct comparison with other systems, the final strength of the loaded APC-XG beads (0.83 N) remained within the general order or magnitude reported for some millimetric phenolic-loaded alginate beads prepared at relatively low polymer concentrations, including barberry extract beads (67–207 g ≈ 0.6–2.0 N) prepared with 0.5–1% alginate [62], grape pomace extract beads (0.29–0.40 N) based on 3% alginate [63], and alginate bigel beads containing EGCG (0.2–0.3 N) prepared with 1% alginate [64]. These findings provide initial evidence of the potential of electrostatic ACP-XG hydrogels to incorporate phenolic compounds. Future research should broaden bioactive incorporation studies by assessing loading process optimization, structural and physicochemical changes following encapsulation and storage, bioactive stability, gastrointestinal behavior, and controlled-release kinetics to comprehensively determine the suitability of these systems for bioactive delivery.

## 3. Conclusions

This study demonstrated the feasibility of producing millimeter-scale hydrogel beads from amaranth proteins and xanthan gum mixtures by extrusion into a glucono δ-lactone bath, offering initial insights into electrostatic gelation under externally acidified conditions. In contrast to previously reported bulk APC-XG electrostatic hydrogels formed by internal acidification, which require several hours to develop, the formation of discrete, self-supporting beads within 10 to 30 min using external acidification represents a substantial reduction in the practical timescale of electrostatic gelation.

In addition to this acceleration, the shift from internal to external acidification led to differences in network development, reflected in the final properties of the beads. Bead size, pH, WRC, syneresis, swelling behavior, gel strength, and the extent of electrostatic crosslinking were influenced by processing conditions, with GDL concentration and hardening time contributing differently depending on the property evaluated.

The results may be explained by a progressive diffusion-driven acidification process, in which proton transport from the surrounding medium is expected to determine the spatial evolution of network formation. However, further research is required to directly elucidate the underlying mechanism, including the characterization of internal pH gradients, charge distribution, and structural evolution during gelation.

Beyond mechanistic insights, APC-XG electrostatic beads showed the ability to incorporate phenolic compounds from coffee pulp. Nevertheless, a systematic evaluation of their encapsulation performance, including stability and release behavior, among others, is required.

This study constitutes an initial step toward understanding electrostatic gelation in protein–polysaccharide systems under external acidification and highlights its potential as an alternative to conventional ionic gelation for fabricating millimeter-scale hydrogel beads.

## 4. Materials and Methods

### 4.1. Materials

An amaranth protein concentrate (APC) from commercial amaranth seeds (*A. hypochondriacus*, CV Revancha) with 74 wt.% protein, 1.1 wt.% fat, 0.1 wt.% fiber, 7 wt.% ash, 3.9 wt.% moisture, and 13.9 wt.% carbohydrates was used; ground coffee pulp (*Coffea arabica* L., CV Typica) was donated from a local producer (Santiago Atitlan, Oaxaca, Mexico); xanthan gum (XG) (G1253, Mw ~2–4 MDa), Folin–Ciocalteau’s phenol reagent, and glucono-δ-lactone (GDL) were purchased from Sigma Aldrich Co. (St. Louis, MO, USA). All other reagent-grade chemicals were acquired from JT Baker (Mexico City, Mexico).

### 4.2. Bead Fabrication

Individual aqueous dispersions of APC and XG (1% *w*/*v*) were prepared with distilled water and magnetically stirred for 3 h at room temperature (25 ± 2 °C) to allow hydration. One hour before the end of this period, the APC dispersion was adjusted to pH 10.0 ± 0.08, and the XG was adjusted to the same pH immediately at the end of the hydration. Then, a protein–polysaccharide mixture was prepared at a 1:1 *v*/*v* ratio (1% *w*/*v* total biopolymer content) [14,15].

Hydrogel beads were produced by extrusion using a KDS100 infusion pump (Kd Scientific, Holliston, MA, USA) at a constant flow of 0.587 mL/min, with a 20 mL-BD Plastipak syringe (Becton Dickinson, S.A., Franklin Lakes, NJ, USA) without a needle. The APC-XG mixture was dropped from a height of 7 cm into a GDL-gelling solution at a biopolymer-to-bath ratio of 1 mL per 40 mL. Under these conditions, 21–22 beads were obtained per batch. The GDL solutions were prepared 24 h in advance and stored at room temperature (25 ± 2 °C) to allow stabilization of the acidifying medium (final pH: 3.02 ± 0.02, 2.72 ± 0.01, and 2.51 ± 0.03, for 1 mg/mL, 2.5 mg/mL, and 5 mg/mL).

The hardening time in the gelling solution was evaluated at 10 and 30 min. After hardening, the beads were recovered and gently rinsed with distilled water to remove residual acid and then characterized further. Samples were coded as GDL concentration × hardening time (e.g., 1–10, for hydrogel beads fabricated with a 1 mg/mL GDL gelling solution and hardened for 10 min).

All parameter levels were selected based on preliminary experiments. In particular, the hardening time, commonly referred to as gelation time in extrusion systems for millimetric bead fabrication, was defined as the residence time required to obtain discrete, self-supporting beads that retained their shape after recovery.

### 4.3. Hydrogel Beads Characterization

#### 4.3.1. Macroscopic Observation, pH, and Size

For macroscopic observation, the APC-XG hydrogel beads were photographed using a cell phone camera [65]. The pH was determined by a destructive method; freshly prepared beads were crushed, the pH was measured with a pH meter (Orion Star A111, Thermo Fisher Scientific, Waltham, MA, USA), and the average diameter of 200 beads was measured using a digital vernier caliper (Traceable^®^, Control Company, Webster, TX, USA).

#### 4.3.2. Intermolecular Forces and FTIR Analyses

The intermolecular forces responsible for hydrogel bead formation were assessed by solubility testing with different solvents, as previously reported [14], with slight modifications. Briefly, fresh hydrogel beads (obtained by dripping 2 mL of the APC-XG mixture) were mixed with 5 mL of the following solvents: HPLC-grade water, pH 8.0 (S1); 0.086 Tris-0.09 M glycine-4 mM Na_2_EDTA, pH 8 (S2); S2 containing 0.5% SDS (S3); and S3 containing 6 M urea (S4). The mixture was homogenized at 14,000 rpm (Ultra Turrax K10, IKA Works Inc., Wilmington, NC, USA) for 1 min and incubated in a thermobath (25 ± 0.5 °C, 60 min, 85 rpm). The soluble protein was then separated by centrifugation (10,000× *g*, 10 min, 25 °C), and protein content was determined by the Bradford method [66]. The contribution of each type of intermolecular interaction to network formation was quantified as the difference in protein solubility between adjacent solvents: S2-S1 (electrostatic interactions), S3-S2 (hydrophobic interactions), and S4-S3 (hydrogen bonding) [67].

Infrared spectra of powdered individual biopolymers and APC-XG beads were recorded over a wavenumber range of 4000–650 cm^−1^ using a Spectrum GX spectrometer (Perkin-Elmer, Waltham, MA, USA) with an ATR accessory. Twenty scans at a resolution of 1 cm^−1^ were acquired. For sample preparation, XG and APC were dispersed in distilled water; adjusted to pH 3, 4, and 5 with 0.1 N NaOH or 0.1 N HCl; stirred for 30 min; and lyophilized. The beads were prepared as described in Section 4.2 and then dried in an oven at 30 ± 2 °C for 12 h. Using the FTIR spectra expressed as absorbance or transmittance, deconvolution and second-derivative analyses were performed to identify changes in protein secondary structure. Deconvolution of the amide I region was performed by Gaussian curve fitting using Origin 8.5 software (Origin Lab Corp., Northampton, MA, USA), and the relative secondary structure content was estimated from peak area percentages according to band assignments reported in the literature [68].

#### 4.3.3. Water Retention Capacity (WRC)

The moisture content of the freshly prepared beads was determined according to AOAC Official Method 925.10 [69]. Prior to measurement, residual surface water was removed using filter paper. The WRC value (g H_2_O/g biopolymer) was calculated from the measured moisture content.

#### 4.3.4. Syneresis

The syneresis percentage of beads was determined according to Wei et al. [70], with some modifications. Residual water from freshly prepared beads was removed with filter paper, and 22 beads were placed in 20 mL glass vials at room temperature (25 ± 2 °C) and weighed. The weight of the expelled water was determined at 1 h intervals over 6 h by removing the expelled water with filter paper strips and reweighing the vials. The syneresis percentage was calculated according to the following equation:

Syneresis = A/A_0_ × 100, (1)

where A_0_ is the initial weight of the water in the gel (g), and A is the weight of the expelled water (g).

#### 4.3.5. Swelling Index (S.I.)

The swelling capacity of dried beads (30 ± 2 °C, 24 h) was evaluated following the methods of Li et al. [71] and Pourkhatoun et al. [72], with modifications. Samples were placed in 100 mL of distilled water and allowed to swell for 6 h (25 ± 2 °C). The initial weight of the beads and the weights at 1 h intervals were recorded. The swelling index was calculated as the percentage increase in the mass of the gel:

S.I. (%) = (w_t_ − w_0_)/w_0_ (2)

where w_0_ is the initial weight of the dry gel (g), and w_t_ is the weight of the beads at time t (g).

#### 4.3.6. SEM

For SEM observation, beads were dried (6 h, 25 ± 2 °C) and mounted on double-sided carbon tape. They were then coated with a 50 nm gold layer in a metal ionizer, and photographic images were captured at 500× and 2500× magnifications. The samples were examined on an EVO-50 microscope (Carl Zeiss, Jena, Germany) at an accelerating voltage of 10 kV.

#### 4.3.7. Compression Test

The strength of the freshly prepared hydrogel beads was measured using a TA-TX2 plus texture analyzer (Stable MicroSystems, Surrey, UK) with a cylindrical probe (D = 15 mm; h = 10 mm) and a Teflon ring (7.89 mm external diameter, 2.65 mm internal diameter, 0.5 mm height) to prevent slipping during the test. The beads were compressed to 40% with a trigger force of 3 g, using 1, 0.5, and 1 mm/s velocities for pre-test, test, and post-test, respectively. Gel strength was defined as the maximum compression force reached during 40% deformation.

### 4.4. Bioactive Loading Experiment

An aqueous coffee pulp extract (CPE) was prepared from powdered pulp (mesh #60) as described by Duangjai et al. [73]. Briefly, a hot aqueous pulp dispersion (1:10 *w*/*v*) at 92 °C was prepared, stirred for 2 min, and cooled in an ice bath to 25 °C. The extract was obtained by centrifugation at 3500 rpm (10 min, 25 °C). Total phenol content was determined in CPE and in CPE after hydrogel loading using the Folin–Ciocalteu method with a gallic acid standard curve [74].

Freshly prepared beads from treatment 1–10 were placed into the CPE at a 1:20 ratio and stirred at 75 rpm for 6 h in a thermobath at 25 ± 0.5 °C. The beads were recovered and rinsed with distilled water. Total phenolic content was determined as the difference between the extract before and after hydrogel bead loading and calculated as mg GAE/g of fresh beads. Also, FTIR analyses and gel strength were performed as previously described in Section 4.3.2 and Section 4.3.7.

### 4.5. Statistical Analysis

All experiments were conducted at least in triplicate using independent batches. For data analysis, a two-way ANOVA was employed to evaluate the effects of GDL concentration, hardening time, and their interaction. Tukey’s test was used for post hoc comparisons of treatment means. Statistical significance was set at *p* < 0.05, and analyses were performed using JMP^®^ 8.0 software (SAS Institute Inc., Cary, NC, USA). Results are reported as mean ± standard deviation (SD).

## Figures and Tables

**Table 1:** Size and pH of APC-XG hydrogel beads ^1^.

Hardening Time (min)	mg/mL GDL	Diameter (mm)	pH
10	1	3.95 ± 0.34 ^Aa^	4.52 ± 0.11
	2.5	3.57 ± 0.15 ^Ab^	4.21 ± 0.09
	5	3.63 ± 0.16 ^Ab^	3.82 ± 0.16
30	1	3.64 ± 0.19 ^Ba^	3.56 ± 0.12
	2.5	3.07 ± 0.14 ^Bb^	3.34 ± 0.05
	5	3.07 ± 0.19 ^Bb^	3.02 ± 0.14

^1^ Values not joined by the same letter are significantly different (*p* < 0.05). Samples are coded as GDL concentration–hardening time (1/2.5/5 mg/mL GDL-10/30 min). Uppercase superscripts indicate comparisons between hardening times for each GDL concentration. Lowercase superscripts indicate comparisons of GDL concentrations at the same hardening time. APC, amaranth protein concentrate. GDL, glucono-δ-lactone. XG, xanthan gum.

## Data Availability

The original contributions presented in this study are included in the article/Supplementary Materials. Further inquiries can be directed to the corresponding authors.

## Supplementary Materials

The following supporting information can be downloaded at: https://www.mdpi.com/article/10.3390/gels12050406/s1, Figure S1: (**a**) Photograph of APC-XG hydrogel beads fabricated under different GDL concentrations (1, 2.5, 5 mg/mL) and hardening times (10, 30 min). (**b**) Photograph of APC-XG hydrogel beads (1–10 treatment) before and after CPE loading. APC. Amaranth protein concentrate. CPE. Coffee pulp extract. XG. Xanthan gum; Table S1: Effects of GDL concentration, hardening time, and their interaction on physicochemical and structural properties of APC-XG hydrogel beads.

## Abbreviations

The following abbreviations are used in this manuscript:

APC	Amaranth protein concentrate
ATR	Attenuated total reflectance
CPE	Coffee pulp extract
FTIR	Fourier-transform infrared spectroscopy
EGCG	Epicatechin gallate
GAE	Gallic acid equivalent
GDL	Glucono-δ-lactone
pI	Isoelectric point
SEM	Scanning electron microscopy
SDS	Sodium dodecyl sulfate
S.I.	Swelling index
TPC	Total phenolic content
WRC	Water retention capacity
XG	Xanthan gum
