# Supplementary material for: Electrostatic Protein–Polysaccharide Assembly as a Potential Alternative to Ionic Gelation for Millimeter-Scale Hydrogel Beads: Insights into Accelerated Gelation from an Amaranth Protein–Xanthan Gum System
Source: Gels. 2026 May 8;12(5):406. doi: 10.3390/gels12050406 (PMC13206067; doi:10.3390/gels12050406)
Supplement: Supplementary file 1 [file gels-12-00406-s001.zip › gels-4301565-supplementary.pdf]

Electrostatic protein–polysaccharide assembly as a potential alternative to ionic gelation for millimeter-scale hydrogel beads: Insights into accelerated gelation from an amaranth protein–xanthan gum system.

María del Carmen Cortez-Trejo [a], Ramón Román-Doval [b], Lucía Abadía-García [a], Sandra O. Mendoza [a*], Silvia L. Amaya-Llano [a*]

[a] School of Chemistry, Autonomous University of Querétaro, Santiago de Querétaro 76010, Querétaro, Mexico.

[b] Tecnológico Nacional de México, Instituto Tecnológico del Valle de Etla, Oaxaca 68230, Oaxaca, Mexico.

***Corresponding authors**:

Silvia Lorena Amaya Llano: samayal@uaq.mx

Sandra Olimpia Mendoza Díaz: smendoza@uaq.mx

Supplementary Material:

Figure s1. (a) Photograph of APC-XG hydrogel beads fabricated under different GDL concentrations (1, 2.5, 5 mg/mL) and hardening times (10, 30 min). (b) Photograph of APC-XG hydrogel beads (1-10 treatment) before and after CPE loading. APC. Amaranth protein concentrate. CPE. Coffee pulp extract. XG. Xanthan gum.

Table s1. Effects of GDL concentration, hardening time, and their interaction on physicochemical and structural properties of APC-XG hydrogel beads.

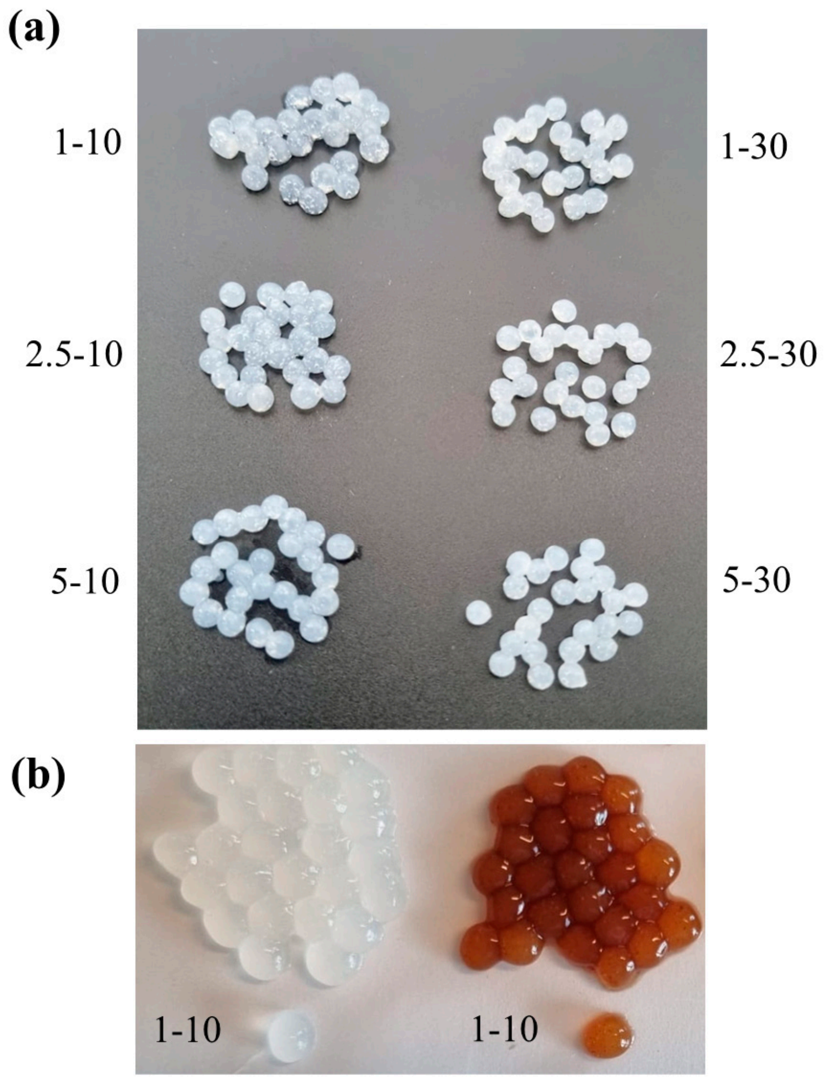

Figure s1. (a) Photograph of APC-XG hydrogel beads fabricated under different GDL concentrations (1, 2.5, 5 mg/mL) and hardening times (10, 30 min). (b) Photograph of APC-XG hydrogel beads (1-10 treatment) before and after CPE loading. APC. Amaranth protein concentrate. CPE. Coffee pulp extract. XG. Xanthan gum.

Table s1. Effects [a] of GDL concentration, hardening time, and their interaction on properties of APC-XG hydrogel beads

Response	Effect	F value	*p*-value
Size	X_1	1.2033	0.3024
	X_2	4.4044	0.0371*
	$X_1{\times}X_2$	3.7469	0.0253*
pH	X_1	794.0244	<.0001*
	X_2	4738.807	<.0001*
	$X_1{\times}X_2$	13.6284	<.0001*
Intermolecular forces:			
Electrostatic	X_1	6.3019	0.0052*
	X_2	47.7904	<.0001*
	$X_1{\times}X_2$	2.4351	0.1047
Hydrophobic	X_1	11.0839	0.0002*
	X_2	1.0397	0.3160
	$X_1{\times}X_2$	11.6380	0.0002*
Hydrogen bonding	X_1	7.0094	0.0032*
	X_2	26.2190	<.0001*
	$X_1{\times}X_2$	0.8822	0.4243
Secondary structure:			
β-sheet	X_1	19.4426	0.0002*
	X_2	0.2758	0.6091
	$X_1{\times}X_2$	8.5575	0.0049*
α-helix	X_1	1.0305	0.3864
	X_2	8.1625	0.0144*
	$X_1{\times}X_2$	3.4928	0.0638
Random coil	X_1	43.7017	<.0001*
	X_2	1.7007	0.2167
	$X_1{\times}X_2$	1.4464	0.2737
β-turn	X_1	9.3312	0.0036*
	X_2	2.0307	0.1796
	$X_1{\times}X_2$	3.5500	0.0615
WRC	X_1	429.0383	<.0001*
	X_2	2278.364	<.0001*
	$X_1{\times}X_2$	53.5221	<.0001*
Syneresis	X_1	3.4655	0.1120
	X_2	17.9684	0.0029*
	$X_1{\times}X_2$	3.1848	0.1141
S.I.	X_1	52.1389	<.0001*
	X_2	158.7808	<.0001*
	$X_1{\times}X_2$	14.0719	0.0007*
Gel strength	X_1	48.7039	<.0001*
	X_2	79.7746	<.0001*
	$X_1{\times}X_2$	11.1872	0.0002*

[a] Results correspond to a two-way analysis of variance (ANOVA) evaluating the effects of GDL concentration (X_1), hardening time (X_2), and their interaction ($X_1 \times X_2$). Significant effects are indicated by * ($p < 0.05$). APC. Amaranth protein concentrate. GDL. Glucono-δ-lactone. S.I. Swelling index. WRC. Water retention capacity. XG. Xanthan gum.
